# Idiopathic Infantile Arterial Calcification: A Possible Cause of Refractory Cardiopulmonary Failure in Infancy

**DOI:** 10.1155/2014/189850

**Published:** 2014-02-09

**Authors:** A. Nael, P. J. Siaghani, D. Chen, S. G. Romansky, L. Shane

**Affiliations:** ^1^Residency Training, Department of Pathology and Laboratory Medicine, University of California Irvine Medical Center, 101 The City Drive, Orange, CA 92868, USA; ^2^University of California, Irvine, Long Beach Memorial Care Health System, 2801 Atlantic Avenue, Long Beach, CA 908068, USA

## Abstract

Idiopathic Infantile Arterial Calcification is a rare autosome recessive disease characterized by extensive calcification of medium and large arteries. Loss-of-function mutations in ectonucleotide pyrophosphatase/phosphodiesterase 1 gene have been described in more than 80% of the cases. Although the diagnosis is usually made at autopsy, it is possible to identify cases based on clinical presentation, radiology findings, and molecular studies. Appropriate treatment can be initiated and has been shown to successfully induce permanent remission. We report a 4-week-old neonate who initially presented with respiratory distress, heart failure, and Coxsackie B viremia suggestive of viral induced cardiomyopathy. His symptoms progressed to multiple organ failure and he eventually expired at four weeks of age. On autopsy, diffuse calcium deposition within the internal elastic lamina of medium and large arteries was identified, as well as narrowing of lumen due to myointimal proliferation. This case report will emphasize the importance of taking this rare curable disease into consideration in all cases of infants with cardiopulmonary failure.

## 1. Introduction

Idiopathic Infantile Arterial Calcification (IIAC) is a rare autosome recessive disease characterized by extensive calcification of medium and large arteries including the aorta, coronary arteries, and renal arteries. Loss-of-function mutation in ectonucleotide pyrophosphatase/phosphodiesterase 1 (*ENPP1*) gene was found in 80% of cases [1]. The disease usually presents from in utero to 3-4 months of age with a wide spectrum of symptoms. The most common constellation of symptoms to raise the possibility of IIAC is respiratory distress, cyanosis, and heart failure [2]. However, rare cases with later presentations have been reported [1, 3]. Without appropriate treatment, IIAC has a high morbidity and mortality rate. On autopsy, calcifications of the large arteries are characteristic, but the most frequent gross findings are myocardial hypertrophy and firm tortuous coronary arteries [1]. Microscopically, diffuse calcium hydroxyapatite deposition within the internal elastic lamina of medium and large arteries with myointimal proliferation and stenosis is characteristic [4, 5].

## 2. Case Presentation

**Figure 1:**
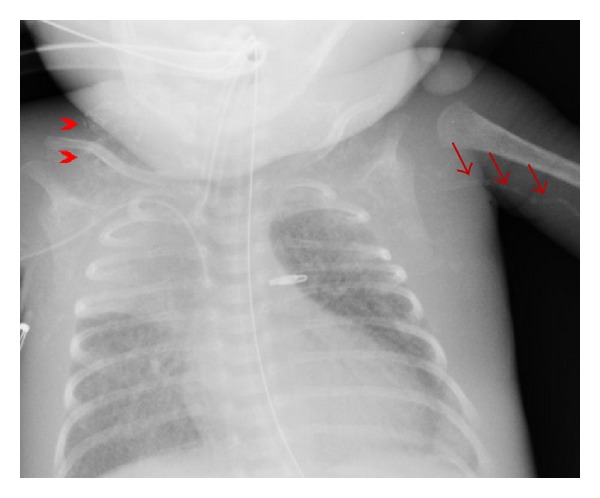
Chest X-ray shows chest soft tissue calcification (arrow heads) and calcification along the left upper arm blood vessels (arrows).

**Figure 2:**
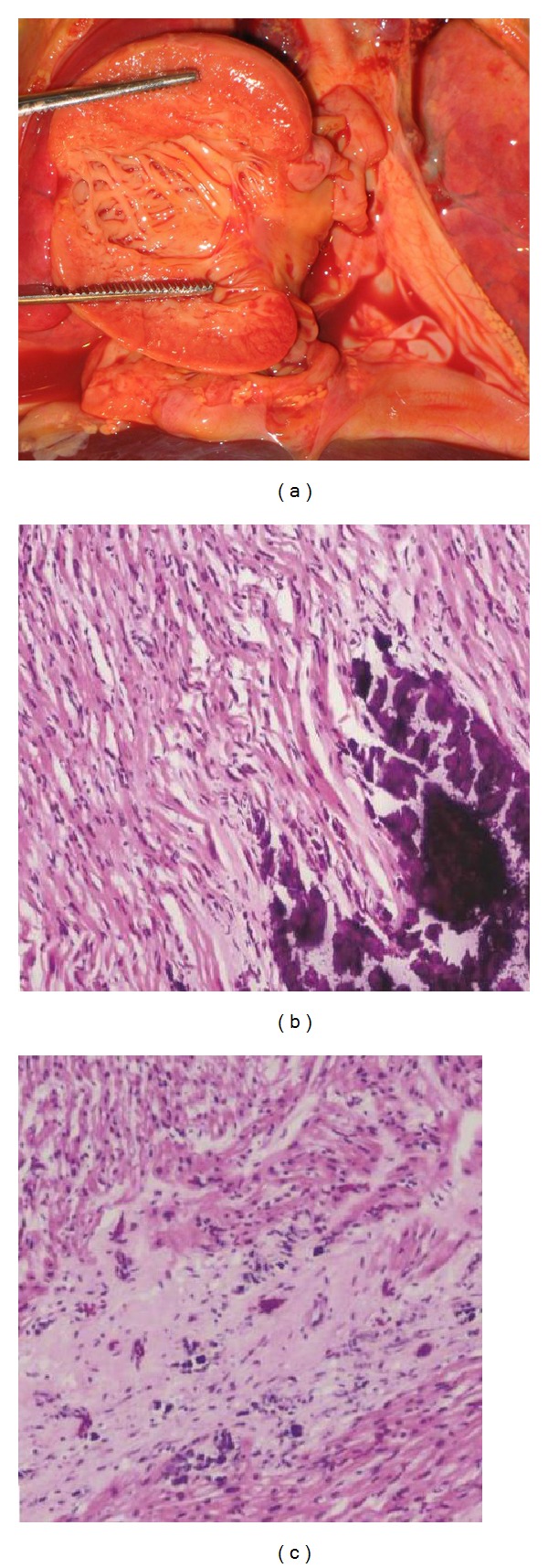
(a) A cut section of the heart demonstrates significant increase in cardiac wall thickness. ((b), (c)) Microscopic section of the heart shows myocardium with extensive calcium deposition (b) and fibrosis (c) with no evidence of inflammation (hematoxylin-eosin, original magnification ×200 [(b), (c)]).

**Figure 3:**
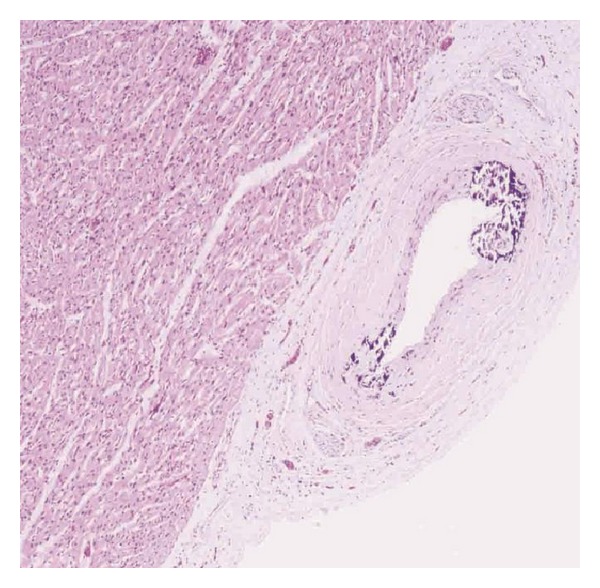
Microscopic picture from the left coronary artery focally demonstrates calcification of the internal elastic lamina (hematoxylin-eosin, original magnification ×200).

**Figure 4:**
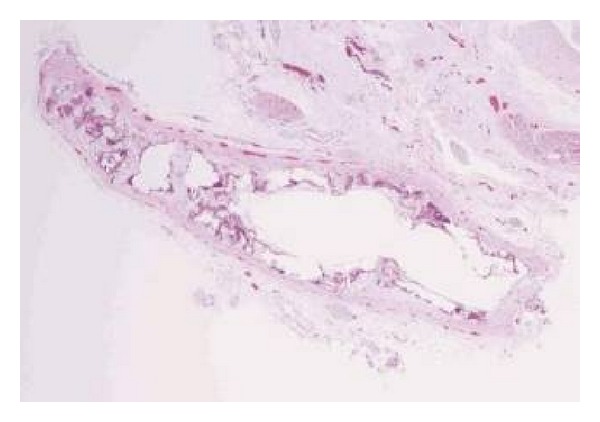
Microscopic picture from the ascending aorta shows extensive calcification with disruption of the elastic lamina and intimal fibrosis (hematoxylin-eosin, original magnification ×200).

**Figure 5:**
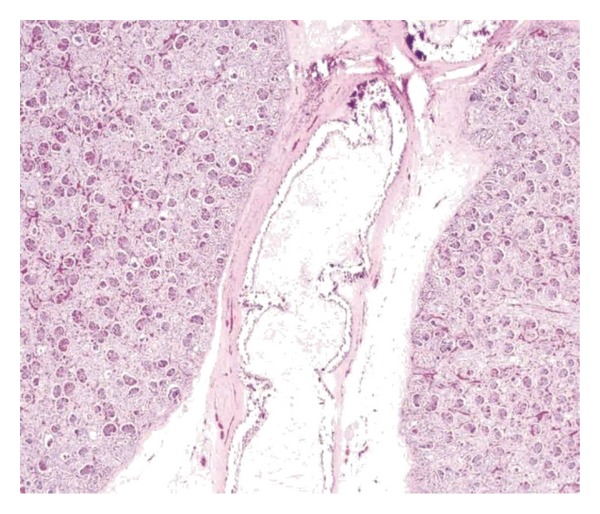
Microscopic picture from the renal hilum shows extensive calcium deposition involving internal elastic lamina with ingrowth of loose fibromyxoid tissue resulting in luminal narrowing and obstruction (hematoxylin-eosin, original magnification ×200).

The patient was a 4-week-old male infant born at an estimated gestational age of 30 weeks with a birth weight of 1665 grams and APGAR scores of 8 and 9 at one and five minutes, respectively. Gross congenital anomalies were not identified. The mother was a gravida 3, para 2, abortion 1, 21-year-old African American with an adequate prenatal care. Her pregnancy was complicated by group b streptococcus urinary tract infection and placental abruption requiring pregnancy termination. In addition, there was a history of hand-foot-mouth syndrome in his sibling two weeks prior to delivery. Immediately after birth, the neonate developed respiratory distress and cyanosis requiring intubation and admission to the neonatal intensive care unit. The initial physical exam revealed a grade III-IV holosystolic murmur and generalized edema. The initial X-rays and echocardiographs demonstrated a moderate to large patent ductus arteriosus (PDA), moderate to severe pulmonary hypertension, and biventricular changes suggestive of hypertrophic cardiomyopathy. Bedside PDA ligation was performed. Subsequent studies revealed Coxsackie B viremia (confirmed by polymerase chain reaction (PCR)); however, TORCH serology were all negative. Viral induced hypertrophic cardiomyopathy was the presumptive diagnosis. However, daily chest X-rays demonstrated progressive nonspecific calcification of the chest soft tissue and the vessels of both arms (Figure 1). Despite multiple intravenous immunoglobulin treatments, loop diuretics, phosphodiesterase 3 inhibitor, and dopamine for the presumed viral induced cardiomyopathy, his symptoms progressed and the patient expired at 4 weeks of age secondary to intraventricular brain hemorrhage and multiorgan failure. At autopsy, there was severe anasarca, but gross congenital anomalies were not identified. Cardiovascular dissection revealed increased biventricular wall thickness (Figure 2(a)) with evidence of gross arterial calcification along the large vessels. Microscopic sections of the heart demonstrated cardiac myocyte nuclei enlargement consistent with hypertrophy and focal calcium deposition and fibrosis within the myocardium. However, inflammation suggestive of myocarditis could not be identified (Figures 2(b) and 2(c)). The coronary arteries demonstrated focal calcification of the internal elastic lamina (Figure 3), while sections from the ascending and descending aorta exhibited extensive calcification with disruption of the elastic lamina and intimal fibrosis (Figure 4). Other organs including lungs, brain, pancreas, kidneys, skin, muscles, and testicles also displayed extensive calcium deposition involving the internal elastic lamina with ingrowth of loose fibromyxoid tissue resulting in luminal narrowing and obstruction. The renal hilar vessels were extensively involved (Figure 5). The gross and microscopic features were consistent with the diagnosis of IIAC. In this case, Coxsackie B viremia and a history of hand-foot-mouth syndrome in the sibling as the etiology of the cardiopulmonary failure were a red herring. However, the single nucleotide polymorphism microarray analysis indicated no clinically significant abnormality and was consistent with a normal male chromosome complement, but the gene sequencing to evaluate the presence or absence of *ENPP1* gene mutation was not performed. This case report aims to emphasize the importance of taking into consideration this rare but curable disease in infants presenting with refractory cardiopulmonary failure.

## 3. Discussion

Although most cases of IIAC are diagnosed at autopsy, there have been reports of diagnosis prior to death based on characteristic findings seen on imaging studies. These include arterial calcification, thickened myocardium, and aortic ring appearance caused by aortic wall calcification [6]. Furthermore, extravascular calcifications in the periarticular cartilage of large joints have been reported in many affected infants [7, 8]. Moreover, a few cases of IIAC discovered on prenatal ultrasound have also been reported by identifying signs of hydrops fetalis and cardiomegaly [9, 10]. Although infancy vascular calcifications are not unique to IIAC and other more common conditions such as hypervitaminosis D, parathyroid abnormalities, intrauterine infections, progeria, Williams's syndrome, pseudoxanthoma elasticum, and Singleton-Merten syndrome should also be kept in mind [2]. However, these cases have different clinical presentations compared to IIAC. In our case the most challenging differential diagnosis was intrauterine fetal infection. Although each of the congenital intrauterine infections has distinct clinical manifestations and sequelae, some of them do share certain clinical features including microcephaly, intracranial calcifications, rash, intrauterine growth restriction, jaundice, hepatosplenomegaly, elevated transaminases, and thrombocytopenia [11]. None of which were present in our case. This in addition to the negative TORCH serology makes the classic group of congenitally acquired infections unlikely. Although Coxsackie B viremia was confirmed by PCR and presumed to be the etiology of the cardiomyopathy, microscopic sections on autopsy failed to demonstrate any evidence of inflammation and thus not supporting viral induced cardiomyopathy as the etiology of the cardiopulmonary failure. Cases clinically suspicious for IIAC can be confirmed with imaging studies, arterial biopsy, and molecular analysis [7, 12]. Early appropriate diagnosis would allow treatment with bisphosphonates, which have been shown to induce permanent remission by inhibiting disease progression and solubilizing calcium deposits with long-term survival [7, 13–16]. The tendency of the disease to occur in siblings raised the prospect of genetic linkage, which has been supported by loss-of-function mutation affecting the *ENPP1* gene with autosomal recessive transmission. The gene encoding *ENPP1* spans 83 kb of genomic DNA and contains 25 exons [8]. About forty different mutations either in homozygote or compound heterozygote have been identified in the *ENPP1* gene linked to IIAC [7]. Rutsch et al. [7, 8, 17] demonstrated that extracellular matrix calcification in IIAC is a default process due to deficiency in inorganic pyrophosphate (PPi). PPi is known as one of the major inhibitors of hydroxyapatite crystal deposition and chondrogenesis in bone and cartilage. In summary, PPi level in plasma and urine is regulated by multiple pathways including *ENPP1* gene function. *ENPP1* gene mutation leads to decreased activity in its downstream product, nucleotide pyrophosphatase 1 (NPP1). Deficiency of NPP1 activity will lead to less PPi generation. Bisphosphonates are PPi analogues, which would explain reports of remission in cases of IIAC treated with bisphosphonate therapy. Although not all cases have this mutation, once the diagnosis is made, the family should be informed and genetic counseling offered in order to initiate treatment in any other surviving family members with IIAC [12, 15, 18]. Moreover, future pregnancies may require prenatal fetal ultrasound as well as molecular studies to aid in the diagnosis of IIAC in affected families with or without the *ENPP1* gene mutation [9].
